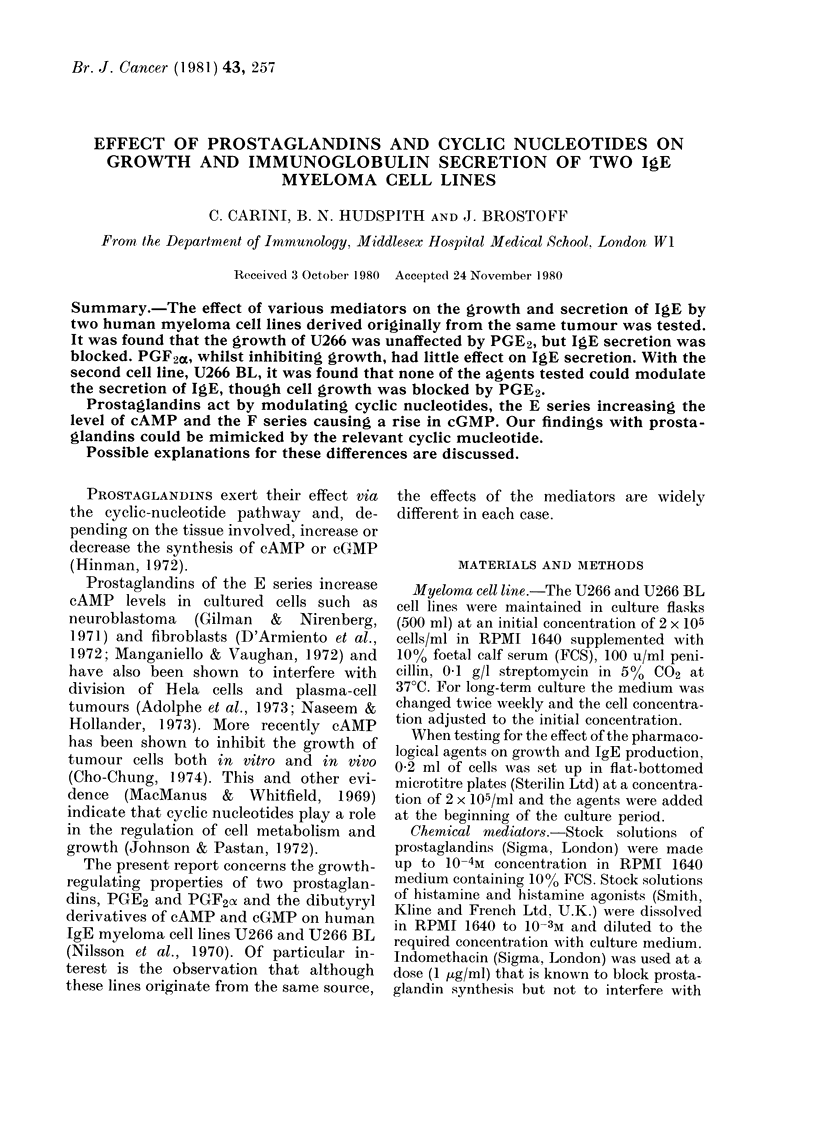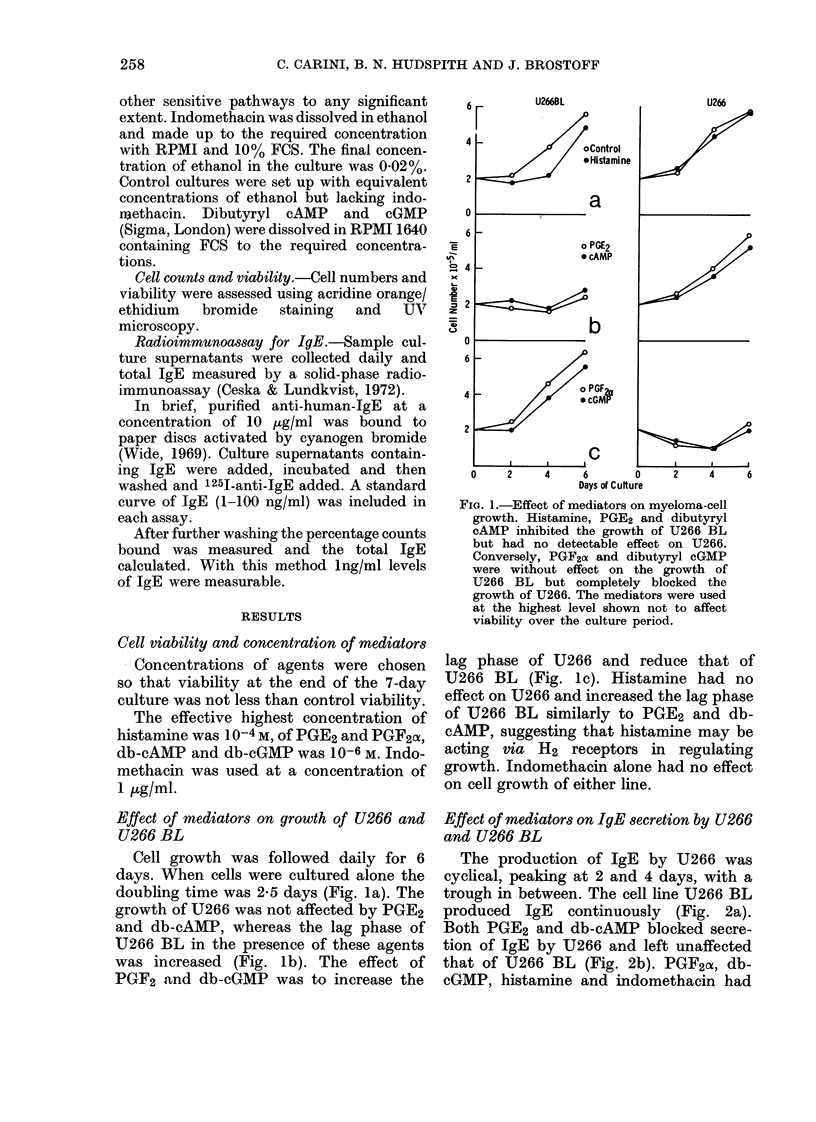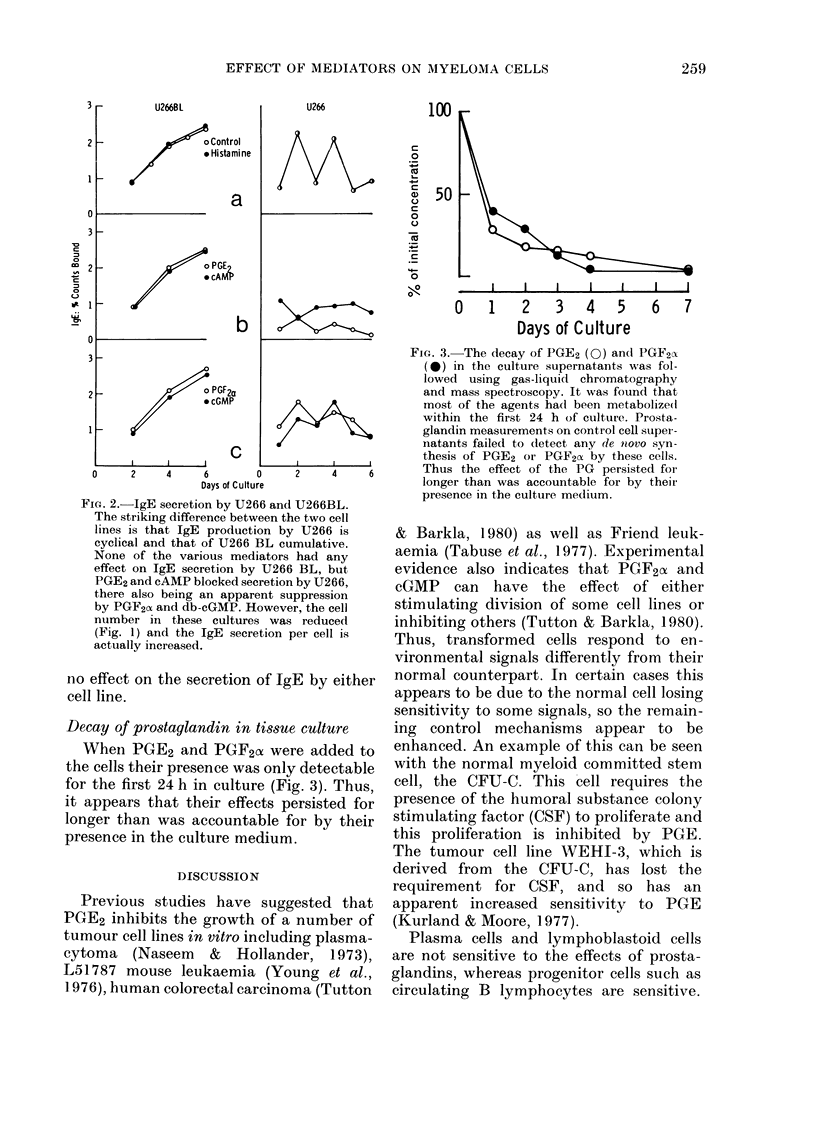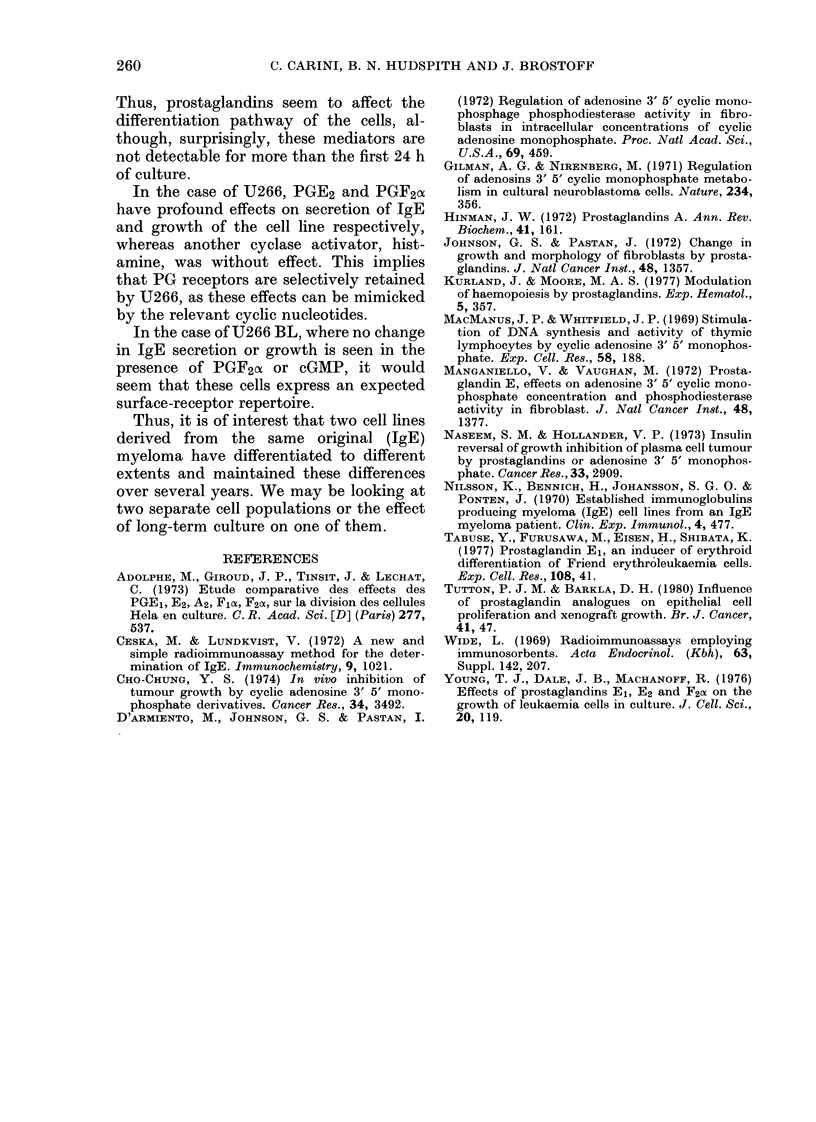# Effect of prostaglandins and cyclic nucleotides on growth and immunoglobulin secretion of two IgE myeloma cell lines.

**DOI:** 10.1038/bjc.1981.42

**Published:** 1981-03

**Authors:** C. Carini, B. N. Hudspith, J. Brostoff

## Abstract

The effect of various mediators on the growth and secretion of IgE by two human myeloma cell lines derived originally from the same tumour was tested. It was found that the growth of U266 was unaffected by PGE2, but IgE secretion was blocked. PGF2 alpha, whilst inhibiting growth, had little effect on IgE secretion. With the second cell line, U266 BL, it was found that none of the agents tested could modulate the secretion of IgE, though cell growth was blocked by PGE2. Prostaglandins act by modulating cyclic nucleotides, the E series increasing the level of cAMP and the F series causing a rise in cGMP. Our findings with prostaglandins could be mimicked by the relevant cyclic nucleotide. Possible explanations for these differences are discussed.


*Br. J. Cancer* (1981) **43**, 257

### EFFECT OF PROSTAGLANDINS AND CYCLIC NUCLEOTIDES ON GROWTH AND IMMUNOGLOBULIN SECRETION OF TWO IgE MYELOMA CELL LINES

C. CARINI, B. N. HUDSPITH AND J. BROSTOFF

*From the Department of Immunology, Middlesex Hospital Medical School, London W1*

Received 3 October 1980 Accepted 24 November 1980

**Summary.—The effect of various mediators on the growth and secretion of IgE by two human myeloma cell lines derived originally from the same tumour was tested. It was found that the growth of U266 was unaffected by $PGE_2$, but IgE secretion was blocked. $PGF_{2\alpha}$, whilst inhibiting growth, had little effect on IgE secretion. With the second cell line, U266 BL, it was found that none of the agents tested could modulate the secretion of IgE, though cell growth was blocked by $PGE_2$.**

**Prostaglandins act by modulating cyclic nucleotides, the E series increasing the level of cAMP and the F series causing a rise in cGMP. Our findings with prostaglandins could be mimicked by the relevant cyclic mucleotide.**

**Possible explanations for these differences are discussed.**

PROSTAGLANDINS exert their effect *via* the cyclic-nucleotide pathway and, depending on the tissue involved, increase or decrease the synthesis of cAMP or cGMP (Hinman, 1972).

Prostaglandins of the E series increase cAMP levels in cultured cells such as neuroblastoma (Gilman & Nirenberg, 1971) and fibroblasts (D'Armiento *et al.*, 1972; Manganiello & Vaughan, 1972) and have also been shown to interfere with division of Hela cells and plasma-cell tumours (Adolphe *et al.*, 1973; Naseem & Hollander, 1973). More recently cAMP has been shown to inhibit the growth of tumour cells both *in vitro* and *in vivo* (Cho-Chung, 1974). This and other evidence (MacManus & Whitfield, 1969) indicate that cyclic nucleotides play a role in the regulation of cell metabolism and growth (Johnson & Pastan, 1972).

The present report concerns the growth-regulating properties of two prostaglandins, $PGE_2$ and $PGF_{2\alpha}$ and the dibutyryl derivatives of cAMP and cGMP on human IgE myeloma cell lines U266 and U266 BL (Nilsson *et al.*, 1970). Of particular interest is the observation that although these lines originate from the same source, the effects of the mediators are widely different in each case.

### MATERIALS AND METHODS

*Myeloma cell line.*—The U266 and U266 BL cell lines were maintained in culture flasks (500 ml) at an initial concentration of $2 \times 10^5$ cells/ml in RPMI 1640 supplemented with 10% foetal calf serum (FCS), 100 u/ml penicillin, 0·1 g/l streptomycin in 5% $CO_2$ at 37°C. For long-term culture the medium was changed twice weekly and the cell concentration adjusted to the initial concentration.

When testing for the effect of the pharmacological agents on growth and IgE production, 0·2 ml of cells was set up in flat-bottomed microtitre plates (Sterilin Ltd) at a concentration of $2 \times 10^5$/ml and the agents were added at the beginning of the culture period.

*Chemical mediators.*—Stock solutions of prostaglandins (Sigma, London) were made up to $10^{-4}$M concentration in RPMI 1640 medium containing 10% FCS. Stock solutions of histamine and histamine agonists (Smith, Kline and French Ltd, U.K.) were dissolved in RPMI 1640 to $10^{-3}$M and diluted to the required concentration with culture medium. Indomethacin (Sigma, London) was used at a dose (1 $\mu$g/ml) that is known to block prostaglandin synthesis but not to interfere with other sensitive pathways to any significant extent. Indomethacin was dissolved in ethanol and made up to the required concentration with RPMI and 10% FCS. The final concentration of ethanol in the culture was 0·02%. Control cultures were set up with equivalent concentrations of ethanol but lacking indomethacin. Dibutyryl cAMP and cGMP (Sigma, London) were dissolved in RPMI 1640 containing FCS to the required concentrations.

*Cell counts and viability.*—Cell numbers and viability were assessed using acridine orange/ethidium bromide staining and UV microscopy.

*Radioimmunoassay for IgE.*—Sample culture supernatants were collected daily and total IgE measured by a solid-phase radioimmunoassay (Ceska & Lundkvist, 1972).

In brief, purified anti-human-IgE at a concentration of 10 μg/ml was bound to paper discs activated by cyanogen bromide (Wide, 1969). Culture supernatants containing IgE were added, incubated and then washed and $^{125}$I-anti-IgE added. A standard curve of IgE (1–100 ng/ml) was included in each assay.

After further washing the percentage counts bound was measured and the total IgE calculated. With this method 1ng/ml levels of IgE were measurable.

FIG. 1.—Effect of mediators on myeloma-cell growth. Histamine, $PGE_2$ and dibutyryl cAMP inhibited the growth of U266 BL but had no detectable effect on U266. Conversely, $PGF_{2\alpha}$ and dibutyryl cGMP were without effect on the growth of U266 BL but completely blocked the growth of U266. The mediators were used at the highest level shown not to affect viability over the culture period.

### RESULTS

### *Cell viability and concentration of mediators*

Concentrations of agents were chosen so that viability at the end of the 7-day culture was not less than control viability.

The effective highest concentration of histamine was $10^{-4}$ M, of $PGE_2$ and $PGF_{2\alpha}$, db-cAMP and db-cGMP was $10^{-6}$ M. Indomethacin was used at a concentration of 1 μg/ml.

### *Effect of mediators on growth of U266 and U266 BL*

Cell growth was followed daily for 6 days. When cells were cultured alone the doubling time was 2·5 days (Fig. 1a). The growth of U266 was not affected by $PGE_2$ and db-cAMP, whereas the lag phase of U266 BL in the presence of these agents was increased (Fig. 1b). The effect of $PGF_2$ and db-cGMP was to increase the lag phase of U266 and reduce that of U266 BL (Fig. 1c). Histamine had no effect on U266 and increased the lag phase of U266 BL similarly to $PGE_2$ and db-cAMP, suggesting that histamine may be acting *via* $H_2$ receptors in regulating growth. Indomethacin alone had no effect on cell growth of either line.

### *Effect of mediators on IgE secretion by U266 and U266 BL*

The production of IgE by U266 was cyclical, peaking at 2 and 4 days, with a trough in between. The cell line U266 BL produced IgE continuously (Fig. 2a). Both $PGE_2$ and db-cAMP blocked secretion of IgE by U266 and left unaffected that of U266 BL (Fig. 2b). $PGF_{2\alpha}$, db-cGMP, histamine and indomethacin had

FIG. 2.—IgE secretion by U266 and U266BL. The striking difference between the two cell lines is that IgE production by U266 is cyclical and that of U266 BL cumulative. None of the various mediators had any effect on IgE secretion by U266 BL, but $PGE_2$ and cAMP blocked secretion by U266, there also being an apparent suppression by $PGF_{2\alpha}$ and db-cGMP. However, the cell number in these cultures was reduced (Fig. 1) and the IgE secretion per cell is actually increased.

FIG. 3.—The decay of $PGE_2$ (○) and $PGF_{2\alpha}$ (●) in the culture supernatants was followed using gas-liquid chromatography and mass spectroscopy. It was found that most of the agents had been metabolized within the first 24 h of culture. Prostaglandin measurements on control cell supernatants failed to detect any *de novo* synthesis of $PGE_2$ or $PGF_{2\alpha}$ by these cells. Thus the effect of the PG persisted for longer than was accountable for by their presence in the culture medium.

no effect on the secretion of IgE by either cell line.

*Decay of prostaglandin in tissue culture*

When $PGE_2$ and $PGF_{2\alpha}$ were added to the cells their presence was only detectable for the first 24 h in culture (Fig. 3). Thus, it appears that their effects persisted for longer than was accountable for by their presence in the culture medium.

### DISCUSSION

Previous studies have suggested that $PGE_2$ inhibits the growth of a number of tumour cell lines *in vitro* including plasmacytoma (Naseem & Hollander, 1973), L51787 mouse leukaemia (Young *et al.*, 1976), human colorectal carcinoma (Tutton & Barkla, 1980) as well as Friend leukaemia (Tabuse *et al.*, 1977). Experimental evidence also indicates that $PGF_{2\alpha}$ and cGMP can have the effect of either stimulating division of some cell lines or inhibiting others (Tutton & Barkla, 1980). Thus, transformed cells respond to environmental signals differently from their normal counterpart. In certain cases this appears to be due to the normal cell losing sensitivity to some signals, so the remaining control mechanisms appear to be enhanced. An example of this can be seen with the normal myeloid committed stem cell, the CFU-C. This cell requires the presence of the humoral substance colony stimulating factor (CSF) to proliferate and this proliferation is inhibited by PGE. The tumour cell line WEHI-3, which is derived from the CFU-C, has lost the requirement for CSF, and so has an apparent increased sensitivity to PGE (Kurland & Moore, 1977).

Plasma cells and lymphoblastoid cells are not sensitive to the effects of prostaglandins, whereas progenitor cells such as circulating B lymphocytes are sensitive.

Thus, prostaglandins seem to affect the differentiation pathway of the cells, although, surprisingly, these mediators are not detectable for more than the first 24 h of culture.

In the case of U266, $PGE_2$ and $PGF_{2\alpha}$ have profound effects on secretion of IgE and growth of the cell line respectively, whereas another cyclase activator, histamine, was without effect. This implies that PG receptors are selectively retained by U266, as these effects can be mimicked by the relevant cyclic nucleotides.

In the case of U266 BL, where no change in IgE secretion or growth is seen in the presence of $PGF_{2\alpha}$ or cGMP, it would seem that these cells express an expected surface-receptor repertoire.

Thus, it is of interest that two cell lines derived from the same original (IgE) myeloma have differentiated to different extents and maintained these differences over several years. We may be looking at two separate cell populations or the effect of long-term culture on one of them.

### REFERENCES

ADOLPHE, M., GIROUD, J. P., TINSIT, J. & LECHAT, C. (1973) Etude comparative des effects des $PGE_1$, $E_2$, $A_2$, $F_{1\alpha}$, $F_{2\alpha}$, sur la division des cellules Hela en culture. *C.R. Acad. Sci.* [*D*] (*Paris*) **277**, 537.

CESKA, M. & LUNDKVIST, V. (1972) A new and simple radioimmunoassay method for the determination of IgE. *Immunochemistry*, **9**, 1021.

CHO-CHUNG, Y. S. (1974) *In vivo* inhibition of tumour growth by cyclic adenosine 3′ 5′ monophosphate derivatives. *Cancer Res.*, **34**, 3492.

D'ARMIENTO, M., JOHNSON, G. S. & PASTAN, I. (1972) Regulation of adenosine 3′ 5′ cyclic monophosphage phosphodiesterase activity in fibroblasts in intracellular concentrations of cyclic adenosine monophosphate. *Proc. Natl Acad. Sci., U.S.A.*, **69**, 459.

GILMAN, A. G. & NIRENBERG, M. (1971) Regulation of adenosins 3′ 5′ cyclic monophosphate metabolism in cultural neuroblastoma cells. *Nature*, **234**, 356.

HINMAN, J. W. (1972) Prostaglandins A. *Ann. Rev. Biochem.*, **41**, 161.

JOHNSON, G. S. & PASTAN, J. (1972) Change in growth and morphology of fibroblasts by prostaglandins. *J. Natl Cancer Inst.*, **48**, 1357.

KURLAND, J. & MOORE, M. A. S. (1977) Modulation of haemopoiesis by prostaglandins. *Exp. Hematol.*, **5**, 357.

MACMANUS, J. P. & WHITFIELD, J. P. (1969) Stimulation of DNA synthesis and activity of thymic lymphocytes by cyclic adenosine 3′ 5′ monophosphate. *Exp. Cell. Res.*, **58**, 188.

MANGANIELLO, V. & VAUGHAN, M. (1972) Prostaglandin E, effects on adenosine 3′ 5′ cyclic monophosphate concentration and phosphodiesterase activity in fibroblast. *J. Natl Cancer Inst.*, **48**, 1377.

NASEEM, S. M. & HOLLANDER, V. P. (1973) Insulin reversal of growth inhibition of plasma cell tumour by prostaglandins or adenosine 3′ 5′ monophosphate. *Cancer Res.*, **33**, 2909.

NILSSON, K., BENNICH, H., JOHANSSON, S. G. O. & PONTEN, J. (1970) Established immunoglobulins producing myeloma (IgE) cell lines from an IgE myeloma patient. *Clin. Exp. Immunol.*, **4**, 477.

TABUSE, Y., FURUSAWA, M., EISEN, H., SHIBATA, K. (1977) Prostaglandin $E_1$, an inducer of erythroid differentiation of Friend erythroleukaemia cells. *Exp. Cell. Res.*, **108**, 41.

TUTTON, P. J. M. & BARKLA, D. H. (1980) Influence of prostaglandin analogues on epithelial cell proliferation and xenograft growth. *Br. J. Cancer*, **41**, 47.

WIDE, L. (1969) Radioimmunoassays employing immunosorbents. *Acta Endocrinol.* (*Kbh*), **63**, Suppl. 142, 207.

YOUNG, T. J., DALE, J. B., MACHANOFF, R. (1976) Effects of prostaglandins $E_1$, $E_2$ and $F_{2\alpha}$ on the growth of leukaemia cells in culture. *J. Cell. Sci.*, **20**, 119.